# LightSit: An Unobtrusive Health-Promoting System for Relaxation and Fitness Microbreaks at Work

**DOI:** 10.3390/s19092162

**Published:** 2019-05-09

**Authors:** Xipei Ren, Bin Yu, Yuan Lu, Biyong Zhang, Jun Hu, Aarnout Brombacher

**Affiliations:** Department of Industrial Design, Eindhoven University of Technology, 5600 MB Eindhoven, The Netherlands; b.yu@tue.nl (B.Y.); y.lu@tue.nl (Y.L.); b.zhang@tue.nl (B.Z.); j.hu@tue.nl (J.H.); a.c.brombacher@tue.nl (A.B.)

**Keywords:** office vitality, microbreaks, ambient technology, workplace health promotion

## Abstract

Physical inactivity and chronic stress at work increase the risks of developing metabolic disorders, mental illnesses, and musculoskeletal injuries, threatening office workers’ physical and psychological well-being. Although several guidelines and interventions have been developed to prevent theses subhealth issues, their effectiveness and health benefits are largely limited when they cannot match workday contexts. This paper presents LightSit, a health-promoting system that helps people reduce physically inactive behaviors and manage chronic stress at work. LightSit comprises a sensor mat that can be embedded into an office chair for measuring a user’s sitting posture and heart rate variability and a lighting display that is integrated into a monitor stand to present information unobtrusively, facilitating fitness and relaxation exercises during microbreaks. Following the showroom approach, we evaluated LightSit during a public exhibition at Dutch Design Week 2018. During the eight days of the exhibition, we observed more than 500 sessions of experiences with healthy microbreaks using our prototype. Semistructured interviews were conducted with 50 participants who had office-based jobs and had experienced LightSit. Our qualitative findings indicated the potential benefits of LightSit in facilitating health-promoting behaviors during office work. Based on the insights learned from this study, we discuss the implications for future designs of interactive health-promoting systems.

## 1. Introduction

Nowadays, a fast-paced workstyle causes prolonged sedentary time and increased chronic stress. As a result, a large percentage of office workers are in a state of subhealth. The prevalence of sedentary work has been shown to be the leading cause of muscular disorders and spine overload [1]. Chronic work-related stress harms office workers’ physical and psychological well-being, which may lead to various health problems, such as obesity, diabetes, and cardiovascular diseases [2]. A recent working condition survey by the Netherlands Organization for Applied Scientific Research (2016) demonstrated that lower back pain (1.7% of the total working population) and burnout (1.4%) are the two most common occupational diseases in the Netherlands, which has resulted in almost 4.3 million days of sickness absence every year [3]. These health problems increase the burden on public healthcare. Besides, work-related stress and sedentariness also decrease the productivity and teamwork of an organization or a company.

To tackle such “workstyle” health problems, many strategies have been proposed to facilitate workplace health initiatives, including counseling interventions [4], organizational wellness programs [5], or promotional materials [6], etc. Moreover, previous research has indicated the potential of the rapid advance of ubiquitous sensing technologies and human–computer interaction (HCI) for supporting office vitality. For instance, various nonintrusive sensors can be embedded in the office environment, monitoring the vital signals and activities data of office workers during work [7,8]. Subsequently, HCI technologies, such as persuasive technology [9], exergames [10], and peripheral interaction paradigm [11], can also be widely applied to improve self-awareness and encourage healthy behaviors at work. For health promotion, it is essential to get users to adhere to health interventions until they are habituated into a healthy lifestyle. However, it is challenging for office workers to adopt health interventions at work when these interventions conflict with their established workstyle or overburden their work routine. According to Chung and colleagues [12], the technical features of health-promoting programs should be designed and implemented based on the characteristics of office settings and workday contexts. We found that few current workplace health technologies were intentionally designed to address this challenge, and therefore they have hardly achieved their desired results or efficiency in health promotion [13].

**Figure 1:**
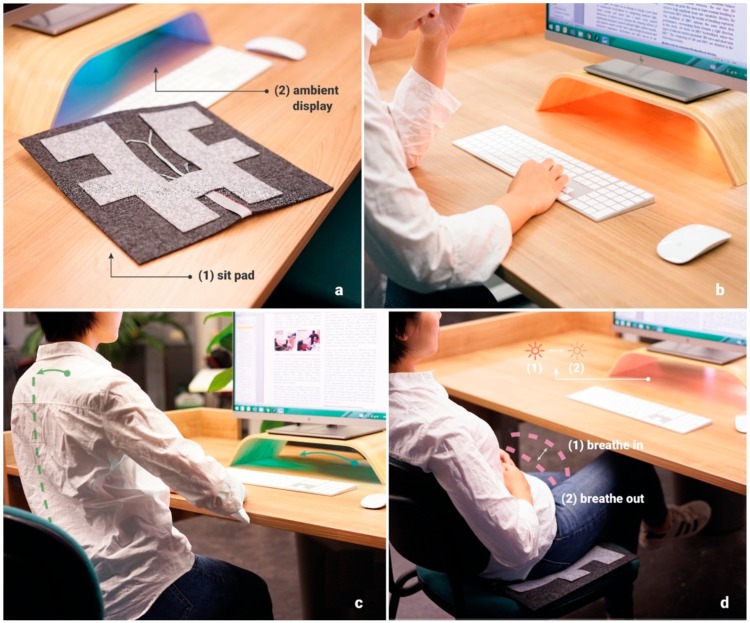
(**a**) LightSit comprises two parts: (1) A sit pad with pressure sensors and (2) an ambient lighting display. (**b**) The peripheral intervention of LightSit: When the user is overstressed and sitting for an extended period, the light becomes brighter and more saturated. (**c**) Stretching fitness: The light waves between left and right to mirror movement. (**d**) Deep breathing relaxation: The light (1) rises and (2) falls to mimic breathing patterns.

In this paper, we present LightSit, an interactive health-promoting system that integrates sensing and HCI technologies into working environments. LightSit aims to provide office workers with an unobtrusive health intervention and support at-the-desk microbreaks that can be interwoven into a work routine. As shown in Figure 1a, LightSit consists of a lightweight sensing pad in an office chair and an ambient display through a light under a monitor stand on the desk. With the sensing pad, the office chair can unobtrusively measure a user’s sitting posture and heart rate variability (HRV) during work. The ambient light can not only visualize sedentary time and stress levels at work to enhance a user’s self-awareness (Figure 1b), but can also present guidance and feedback information to assist with lower back stretching (Figure 1c) and deep breathing (Figure 1d) exercises as micro-interventions interwoven into a work routine. The LightSit system was demonstrated at Dutch Design Week 2018, and its user experience and acceptance were evaluated following the showroom approach [14], in which more than 500 participants experienced our system. Based on observations and participant feedback, we discuss the lessons learned and derive several implications for the future design of workplace health-promoting technologies.

## 2. Health-Promoting Technologies for Office Vitality

In this section, we first provide an overview of related work on sensing technologies for measuring office vitality. We then describe state-of-the-art designs using HCI in interventions to promote health and well-being in the workday context.

### 2.1. Sensing Technologies for Measuring Physical and Mental Health at Work

In workplace health-promoting systems, sensing a user’s physical and mental health status at work is essential and usually the first step in health intervention. Office workers’ physical movements and mental stress can be measured by computer software or various sensors deployed in office environments. Sedentary behavior can be recognized through an analysis of image sequences captured by cameras. For instance, *SuperBreak* implements a computer vision algorithm in a webcam so that it can detect the hand movement of an office worker sitting at a desk [7]. Similarly, Reilly and colleagues (2013) attached a Kinect sensor to a workstation for tracking sitting conditions and arm movements during work [15]. Another activity sensing approach is analyzing motion sensor data based on an actual context to identify specific behaviors related to physical activity. For example, *Exerseat* installs a proximity sensing toolkit on office chairs to monitor whether users are sitting on or near the seat [16]. In addition, Peeters et al. [17] equipped steps of a staircase with pressure sensor strips to track stair climbing behavior. More recently, *StretchArms* [18] acquired accelerometer data from a smartwatch and applied an algorithm to interpret motion data as different degrees of arm stretching.

Besides sedentary behaviors, chronic stress has become increasingly severe in modern work. As stress affects individuals’ autonomic nervous systems, various automatic stress recognition systems have been developed to estimate office workers’ stress levels by monitoring relevant physiological parameters, among which heart rate variability (HRV) is the most commonly used one. HRV has been proven to be a sensitive biomarker [19] in effectively indicating mental stress during computer work [20], driving [21], or mentally challenging tasks [22]. HRV is usually calculated from electrocardiogram (ECG) [23] or blood volume pulse (BVP) signals [24]. The acquisition of both types of signals requires users to continuously wear a piece of equipment with biosensors that are obtrusive for office workers. For instance, ECG acquisition involves a set of electrodes to be attached on a user’s chest [20], and a robust BVP signal is usually measured by a photoplethysmographic (PPG) sensor placed on a fingertip or an earlobe [22]. Given the intrusive nature of ECG and PPG sensors, they may not be ideal approaches to stress measurement at work. Therefore, we argue that it is meaningful to apply a more unobtrusive approach for HRV measurement in everyday office environments.

### 2.2. Ambient HCI for Promoting Health at Work

In recent years, considerable studies in ambient intelligence [25] have been devoted to promoting office vitality and health management. Compared to traditional screen-based displays, ambient human–computer interaction has shown big advantages in reducing an intervention’s interference with ongoing tasks and enhancing a user’s self-awareness of health during work. For instance, Jafarinaimi et al. [11] designed a desktop decorative object representing a user’s health information to encourage physical activities and reduce sedentary time among office workers. Hong et al. [26] designed a flower-like avatar that can change shape to suggest postures to a user and encourage active sitting. Moraveji et al. [27] developed a peripheral respiration training system to facilitate slow breathing during work. Additionally, environmental lighting [28], interactive sound [29], and haptic feedback [30] have also been explored as ambient displays to promote occupational health. These studies have suggested that ambient displays are effective in increasing self-awareness without requiring a user’s full attention. Ambient displays may stimulate health behaviors and deliver health intervention in a more unobtrusive manner [22], which is suitable for a workday context.

Advanced sensing techniques can enrich health monitoring parameters, and human–computer interaction techniques can enhance user experiences. By combining both technologies, an increasing number of interactive health-promoting systems have been developed for improving a user’s engagement with a health intervention. For instance, *Tap-Kick-Click* tracks foot movements and facilitates foot-based interaction using a pair of smart shoes to facilitate healthy postures at work [31]. *Sonic Cradle* creates a meditation environment where the user is immersed in a soundscape responding to their breathing patterns [32]. However, such an interactive health-promoting system is relatively difficult to deploy in office environments for use by office workers in the long term [33]. It has been suggested that workplace health-promoting technologies should be integrated into an office environment and be interwoven into work routines [33].

## 3. Design of LightSit

In this project, we designed LightSit, a health-promoting system that can (1) unobtrusively sense an office worker’s sedentary workstyle and chronic stress and (2) provide intervention and guidance through an ambient lighting display to facilitate fitness and relaxation exercises at work. LightSit serves as a research probe through which we can explore technology-mediated microbreaks for health promotion and investigate user experiences and acceptance toward newly developed unobtrusive sensing and ambient intelligence for workplace use.

In this section, we first introduce design considerations regarding heathy microbreaks and the integration of the system into office environments. Then, we present the technical implementation of the LightSit system, including the hardware design, as well as mechanisms for activity tracking and stress detection. This is followed by a description of our user application design, consisting of an awareness intervention and two interactive health-promoting programs.

### 3.1. Design Considerations

#### 3.1.1 Blend Microbreaks into a Work Routine Involving Desk Exercises

It has been proven that, during busy work, even a short one-minute break (namely, a microbreak) with self-regulated healthful behaviors can benefit an individual’s physical and mental well-being [34]. A number of studies have indicated that a short bout of relaxation at the desk during office work can boost the productivity of employees [35]. In this project, we explored a new interventional strategy through LightSit, which facilitates office workers in doing lower back stretching and deep breathing during microbreaks at a desk and habituates such healthy breaks through repeated exercises in a work routine. We identified a typical scenario for LightSit-enabled breaks: “Gain awareness of unhealthy behaviors during work. Then commit some low-effort desk exercises through self-regulation (e.g., stretching, deep breathing) without leaving the ongoing work.”

#### 3.1.2 Embed Sensing and Feedback Technologies into the Office Environment

LightSit is designed to track an office worker’s health status and promote microbreaks involving fitness and relaxation during work. Therefore, to improve the usability and user experience of the system and to lower interference with workflow, we integrated the sensors and feedback display into workplace facilities. Specifically, we developed a fabric seat pad that can be placed on an office chair to unobtrusively measure a user’s sitting posture and heartbeat during work. We embedded a light strip into a monitor stand as an ambient display to visualize a user’s sedentary time and stress status for an intervention and display guidance information to assist with lower back stretching and deep breathing exercises. In this way, LightSit can be easily blended into conventional workstations.

### 3.2. The Technical Development of LightSit

#### 3.2.1. Sensors and Hardware

**Figure 2:**
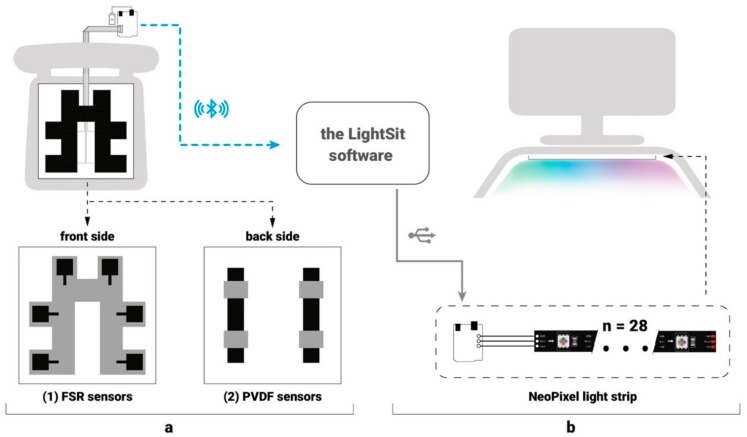
The technical implementation of the LightSit system. (**a**) A seat pad equipped with (1) six FSR sensors and (2) two PVDF sensors. (**b**) A flexible light strip that can be used as an ambient display.

Based on earlier explorations [33,36], we developed a seat pad that can monitor a user’s sitting behavior and heart rate (Figure 2a). Similarly to Reference [37], the pad was designed at a size of 40*40 cm^2^, which ensured that it fits regular office chairs. As shown in Figure 2a, on the front side of the fabric pad, six square-type force sensing resistors (FSRs) [38] are symmetrically distributed on the left and right sides. The combination of multiple FSRs enables the detection of different sitting postures and sedentary durations. The locations of the FSRs were decided based on references from a sedentary pressure map [39]. On the other side of the seat pad, two polyvinylidene fluoride (PVDF) film sensors [40] are used for measuring cardiac vibration signals (also called ballistocardiography (BCG) or a BCG signal) from heartbeats. The BCG signals are then processed and analyzed to calculate the HRV, which has been widely used as a robust stress indicator (e.g., in Reference [22]). All of the data sensed by the seat pad are transmitted to Adafruit METRO M0 Express [41], a 32-bit Arduino-compatible microcontroller, which has a 16-bit analog–digital converter (ADC) and an AT-09 Bluetooth 4.0 module [42] connected. The 16-bit ADC provides enough of a wide dynamic range for BCG data acquisition (from 0 to 4096).

Through the Bluetooth connection, the collected data are then transmitted to software developed based on the *Processing* platform [43]. In the software, the data can be processed into targeted information, which maps to changes in light color and patterns as feedback. As shown in Figure 2b, a flexible light strip with 28 *NeoPixel* RGB LEDs [44] was integrated into the bottom surface of a monitor stand as a lighting display to facilitate ambient user–system interaction and present feedback information beyond screen-based visualizations.

#### 3.2.2. Sedentary Workstyle Tracking

Based on the hardware implementation, we used the data collected from the six FSRs to recognize a user’s sitting postures and variances. Zemp and colleagues [45] demonstrated the feasibility of using various machine learning approaches to classify sitting postures based on data from multiple pressure sensors on the seat pan. Similarly, in this study, a specialized artificial neural network (ANN) was applied to process the sensory data from FSRs for detecting different sitting behaviors of the user. In the LightSit system, we applied a conventional three-layer (an input layer, a hidden layer, an output layer) feedforward network using the Levenberg–Marquardt algorithm, which has been widely applied and validated in a variety of ANN applications, such as greywater treatment [46], skin lesion analysis [47], posture classification [45], etc.

**Figure 3:**
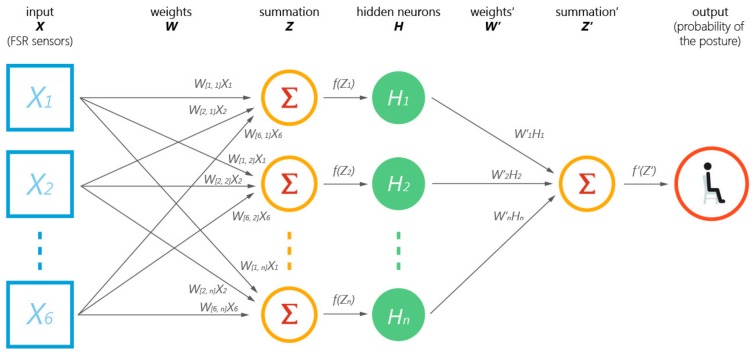
The artificial neural network (ANN) used in LightSit.

As shown in Figure 3, the underlying mechanism of how the ANN works in LightSit can be described as follows. To start, the input data *X* are collected from each of FSR pressure sensors, and weights *W* are given to calculate the summation *Z = ∑ W_[i, n]_ ∗ X_i_*. In the case of LightSit, *i* ∈ {1, 2, 3, 4, 5, 6}, *n* ∈ {1,2, …, the number of hidden neurons}. Then, *Z* is translated into hidden neurons *H*, and the summation of total hidden neurons *Z’* is calculated with weights *W’* as follows: *Z’ = ∑ W’_n_ ∗ H_n_*, where *n* ∈ {1,2, …, the number of hidden neurons}. Finally, *Z’* feeds forward to provide an output that gives a probability of the user’s current sitting condition. In this way, the learning algorithm can be taught iteratively to recognize multiple different postures and calibrate the movement range of weight-shifting according to different users. Based on the application of this ANN mechanism, the LightSit system can analyze a user’s sitting behaviors and customize exercises for active sitting accordingly.

#### 3.2.3. Stress Detection

**Figure 4:**
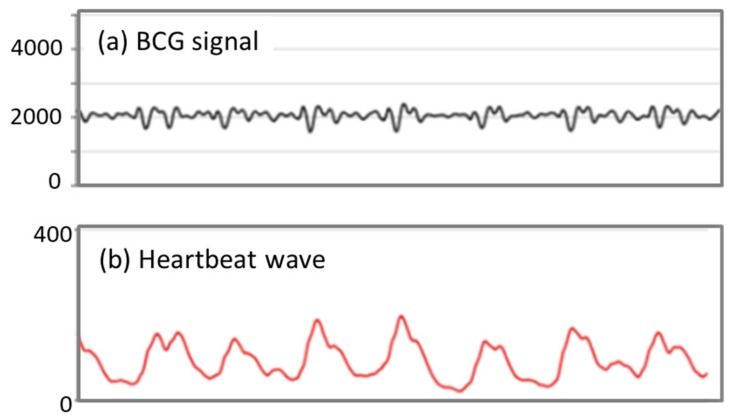
(**a**) Raw ballistocardiography (BCG) signals from an electromechanical film sensor (EMFi), and (**b**) a heartbeat wave extracted by the mean absolute deviation (MAD) algorithm from the BCG signal.

In LightSit, we use two electromechanical film sensors (EMFi) to measure the BCG signal. As shown in Figure 4a, when someone is working without substantial body movements, the BCG signal shows a periodic waveform reflecting the mechanical activity of the heart. BCG signals have been widely studied for heartbeat detection (e.g., References [48,49]). In this study, we developed a robust BCG processing method to calculate the HRV as a physiological measure for stress recognition. The proposed method consists of three major parts: wavelet denoising, interbeat interval (IBI) calculation, and HRV analysis.

First, we applied a wavelet transform to denoise the original BCG signal and remove low-frequency respiratory components. We applied a Haar (“db1”) wavelet transform to the BCG signal and selected a “level 4” detail coefficient to be the denoised signal. Second, we used a moving mean absolute deviation (MAD) to extract the heartbeat signal from the BCG signal. The moving MAD algorithm highlights heartbeat locations by emphasizing the instantaneous amplitude changes of the signal, as shown in Figure 4b. Then, we used the moving maximum algorithm to detect heartbeats. The peak value was detected in a moving window of 400 ms. The heartbeats were located based on the time at which the most significant peak in the window was determined [49]. The time intervals between two adjacent detected heartbeats were calculated as the interbeat interval (IBI) data. This proposed method to detect IBIs for HRV analysis was validated with 10 office workers under different working tasks. We compared the accuracy of the IBI data derived from the LightSit’s BCG signal and an ECG device. The result showed that the average HRV error was 5.77 ms, which confirmed the good performance of the proposed system in HRV calculation. The details of this study can be found in Reference [50].

Finally, we calculated the HRV for estimating a user’s stress. The HRV has a significantly negative correlation with stress. When an individual is exposed to stress for a long period, his/her HRV decreases compared to a normal healthy level. The SDNN (the Standard Deviation of IBI data) is one of the most common HRV indices in the time domain, and it can reflect the overall balance of the autonomic nervous system (ANS), which is closely related to stress responses [19]. A specially modified form of the SDNN was calculated in a moving window of 40 valid IBIs by the formula *HRV*_40_ = ((39 × *HRV*_40_ +|*IBI* − *IBI_40_*|)/40) and *IBI_40_* = (39 × *IBI_40_* + *IBI*)/40. *HRV*_40_ was updated with each heartbeat and mapped to the lighting parameters. Each person has his/her own physiology and HRV range. Therefore, a range of HRV data for each user is required in LightSit system initialization. In our previous study [22], the user first relaxed with slow breathing for one minute, during which the SDNN was calculated as the maximum HRV. Next, the user did one-minute physical exercises. We calculated the SDNN of the following one minute as the minimum HRV. In this way, the HRV range of each user was calculated and could be mapped to the range of lighting parameters for stress intervention.

### 3.3. The Design of Interactive Applications

As mentioned above, LightSit can measure the user’s sitting data and HRV data to calculate the sedentary time and stress level. Based on these measurements, we designed two working modes of LightSit: (1) an ambient intervention mode for prompting microbreaks and (2) an interactive exercise mode for facilitating fitness and relaxation during microbreaks.

#### 3.3.1. The Ambient Intervention Mode

**Figure 5:**
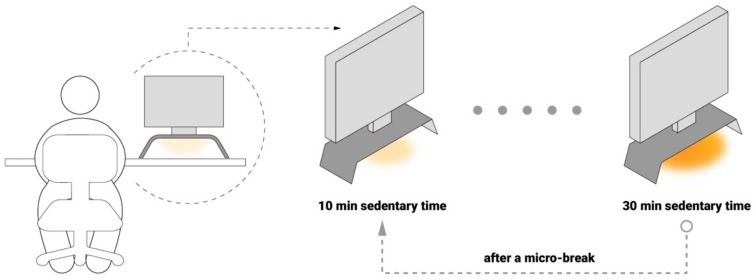
The working mechanism of LightSit as a peripheral intervention.

The accumulated sedentary time and HRV data are visualized through ambient light under a monitor stand. The subtle changes of the light display aim to improve an office worker’s self-awareness of his/her sedentary workstyle and chronic stress but without disturbing ongoing work tasks. For signals for ambient intervention, we mapped prolonged sedentary time (10–30 min) to the brightness of the light (10 to 255) and the HRV value (the maximum to minimum of an individual’s range) to the saturation of the light (10–255). As shown in Figure 5, when the user sits for an extended period with high stress levels (close to minimum HRV value), the light becomes brighter and more saturated (orange).

With improved awareness of sedentary behavior or chronic stress through light changes, office workers may initiate a microbreak, during which they can practice lower back stretching and slow breath training assisted by the interaction mode of LightSit. With this, the prolonged sedentary time is expected to be interrupted, and the HRV is expected to increase. After the healthy microbreak, the lighting display returns to its original state, which is almost invisible in the working context.

#### 3.3.2. The Interactive Exercise Mode

When LightSit detects the user starting lower back stretching or slow breathing for a microbreak, the system switches to the interactive exercise mode automatically. In the interactive exercise mode, LightSit leverages the dynamic changes of the light to provide either motion-based interaction to assist with stretching exercises or to provide HRV biofeedback to facilitate slow breathing.

#### 3.3.3. Assist with Lower Back Stretching

**Figure 6:**
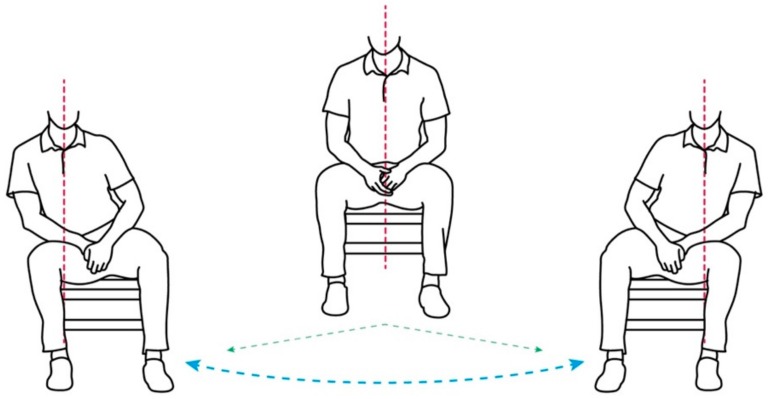
A demonstration of the lower back stretch involved in LightSit.

As shown in Figure 6, LightSit facilitates a lower back stretching exercise, which was adapted from dynamic weight shifting [51]. Specifically, dynamic weight shifting involves slow trunk movements on the pelvis to shift body weight laterally, with a few seconds of stretch hold on each side. As mentioned earlier, musculoskeletal back pain is one of the most critical occupational health issues [52]. Lower back stretching has been evaluated in many other studies as an effective desk exercise in preventing lower back pain for office workers [53,54] (as well as in our earlier work [33]).

**Figure 7:**
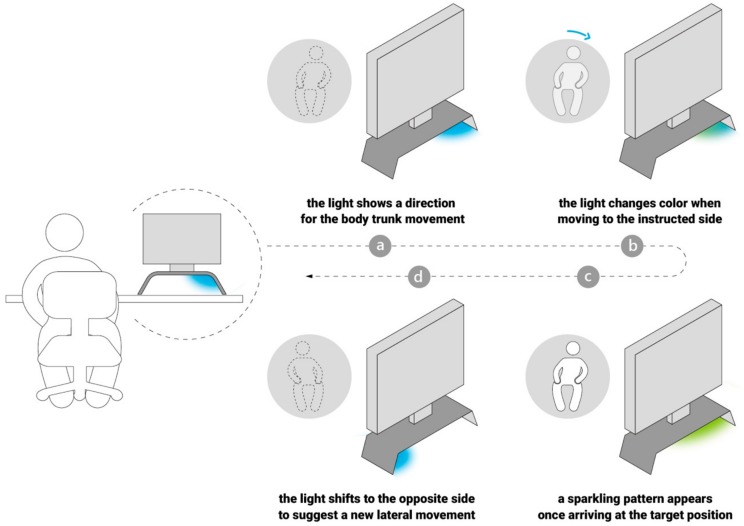
The working mechanism of LightSit to assist with lower back stretching fitness. (**a**) The light shows a direction for the body trunk movement. (**b**) The light changes color when the user moves to the instructed side. (**c**) a sparkling pattern appears once the user arrives at the target position. (**d**) The light shifts to the opposite side to suggest a new lateral movement.

As shown in Figure 7, the lighting display was designed to engage the office worker in the stretch training by providing guidance for achieving target movements and real-time feedback about the exercise flow and results. First, the light is manipulated with a wiping effect [55], which waves between left and right to give cues about lateral movements (see Figure 7a). Second, when the user sways his/her body trunk toward the instructed side, the light gradually changes from a blue color to green in response to the posture changes (see Figure 7b). Third, the light shows a sparkling pattern once the user arrives at the target position (see Figure 7c). To realize the sparkling light pattern, we applied an algorithm based on the ignite effect [56]. After a few seconds of stretching, the light shifts to the next position on the opposite side and changes to the original color of blue (see Figure 7d).

#### 3.3.4. Facilitate Deep Breathing 

**Figure 8:**
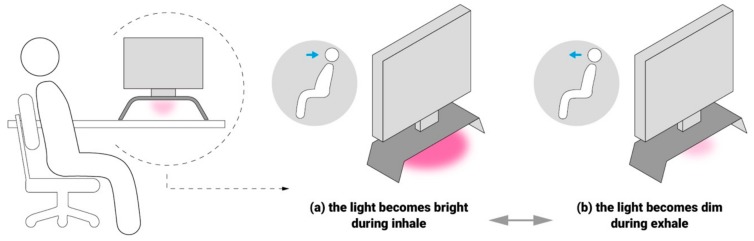
The working mechanism of LightSit to facilitate deep breathing relaxation. (**a**) The light becomes bright during inhale. (**b**) The light becomes dim during exhale.

As shown in Figure 8, when the system recognizes the user sitting still and the HRV increasing rapidly due to slow breathing, LightSit starts providing HRV biofeedback, in which the intensity of the light is directly mapped to real-time heartbeat interval data. HRV biofeedback has been widely documented as an effective tool in mitigating stress and promoting relaxation [22,57,58,59]. In this case, the lighting display serves as an interface for an HRV biofeedback [60] system that shows respiratory sinus arrhythmia. When coupled with heartbeat interval (IBI) data, the light brightness increases and decreases in response to a user’s breathing. Specifically, we mapped IBI data (from 550 to 1150 ms) to the brightness of the light (10 to 255) so that the light becomes bright during inhalation (see Figure 8a) and becomes dim during exhalation (see Figure 8b). This immediate feedback can help the individual better focus on breathing regulation and achieve a smooth and deep breathing pattern. The user can set up a light color that makes them feel relaxed. The LightSit system calculates the user’s *HRV*_40_ and maps it to a saturation value ranging from 10 to 250. Thus, a highly saturated colored light represents an improved HRV, which indicates a healthy benefit from slow breathing.

## 4. The Showroom Evaluation of LightSit

In previous studies, the feasibility of the LightSit system for facilitating microbreaks [33], as well as the effectiveness of ambient lighting for intervening in health behavior and promoting relaxation [22], were evaluated through different lab-based experiments. These evaluations validated the design of LightSit in improving the engagement of office vitality and providing physical and mental benefits. However, these studies were conducted in a controlled office setting with a specific group of university employees, which may restrict the interpretation of our results in a broader population of office workers [61]. Based on the updated prototype, therefore, this study aims to assess the applicability of LightSit to office workers from various working contexts.

We carried out a showroom evaluation [14], where the LightSit system was demonstrated at an international exhibition during Dutch Design Week (DDW) 2018 [62] and was experienced by larger populations with a wider variability in occupations. This showroom evaluation allowed us not only to investigate multiple touchpoint experiences of the newly designed LightSit system with a wide range of potential users, but also to understand the user’s acceptance of the proposed technology-promoted microbreaks at work. First, DDW is featured as the second-largest design exhibition in Europe, and it attracts visitors from not only the Netherlands but also from all over the world. This ensured us easy access to office workers with diverse sociocultural backgrounds. Second, in a showroom practice, the setup of an interactive demo allowed audiences to experience the designed artifacts and communicate their on-the-spot feelings and thoughts with us. Besides, the high quality of the showroom and its inviting ambience could also improve participant involvement in the discussion [63,64]. Therefore, participants became proactive in sharing their insights into our design and research context. Next, we elaborate on the user study and its results in some detail.

### 4.1. The Study

#### 4.1.1. Setup

**Figure 9:**
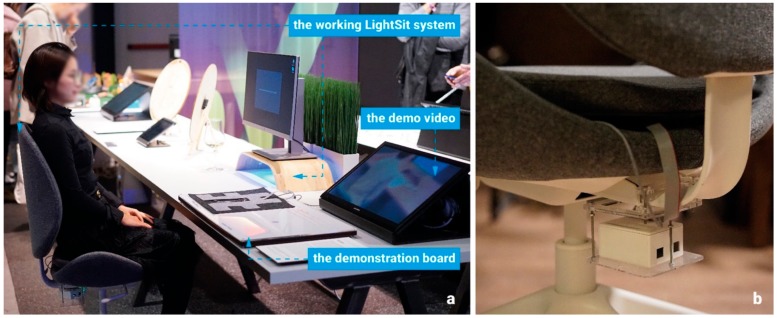
The setup of the exhibition at Dutch Design Week (DDW) 2018.

As shown in Figure 9a, the exhibition of LightSit consisted of a demonstration board to introduce the design and technical implementation of the system, a demo video to illustrate the scenario of using LightSit for improving office vitality, and a working LightSit system that enabled the participants to try it out, performing lower back stretch exercises or slow breathing relaxation sessions. To set up the exhibition, we placed the sensor pad of LightSit into the cushion of an armless office chair and located all of the other electronic components under the seat (see Figure 9b). We installed the LightSit software on a computer, which connected to the lighting display. In the software, we also implemented a graphical interface that invited audiences to try out the LightSit system. The user interface of the LightSit software was presented on a screen placed on top of a wooden stand, which was also equipped with the ambient lighting display. This showroom-based user study lasted for eight days, during which we collected observation data to analyze users’ interactive behaviors during the usage of LightSit and conducted on-the-spot interviews to obtain more detailed feedback. Through these collected data and feedback, first we aimed to understand participants’ user experiences with the desk exercises and relaxation facilitated by the interactive LightSit system. Second, we aimed to learn whether the current design of LightSit applies to workplace health promotion.

#### 4.1.2. Recruitment

During the exhibition, we invited visitors to LightSit to experience the prototype and share their opinions on using the system (in a short follow-up interview). This study did not involve the collection of any physiological data and medical data. During recruiting, we fully informed participants about the procedure of the study and got their consent to take notes on observations of the experience session and the interview. The participants for whom it might have been risky to perform the stretching and deep breathing exercises were excluded. They were also given the opportunity to withdraw at any point. As the study was conducted at an exhibition based on a showroom approach [14], we did not manage to collect participants’ information, such as age and gender. During this eight-day showroom-based study, more than 500 participants tried out the LightSit system. We interviewed 50 participants who had an office-based job just after their experience with LightSit.

#### 4.1.3. Procedure and Data Collection

For each user experience session, the participant performed a short period of lower back stretching and slow breath training using the interactive exercise mode of LightSit. We set the duration of each session as one minute to ensure each participant could receive a full experience delivered by interaction with LightSit, but also to ensure a maximum number of possible participants could experience LightSit. Participants could repeat the exercise or stop at any time. The participants did not experience the intervention mode due to the exhibition setting. Instead, they learned it from the demo video. During the experience session, we observed the participants’ interaction process with the LightSit system and focused on two aspects regarding user experience: (1) How long it would take the participant to understand the ambient feedback and interact with the LightSit system, and (2) how well the participant performed the lower back stretch or deep breathing exercise with LightSit. In this study, two experimenters passively observed experience sessions and took notes for the collection of observational data, which supported a more vibrant representation and understanding of the user experience [65].

After interacting with LightSit, part of the participants were also invited for a short interview, based on their convenience. All of the interviews were semistructured in order to understand the applicability of LightSit to the target context and to find opportunities for further development. Therefore, each session of the interviews was organized into two parts. We began with inquiring about the feasibility of the LightSit-supported interventions by asking questions: “Do you want to use LightSit in your working days? And why?” We then discussed opportunities to improve the design of the system with two open-ended questions: “What did you like most about LightSit?” and “What did you dislike most about LightSit?” During the interview, we left enough space for participants to freely elaborate on their opinions about using LightSit for workplace health promotion. In addition, we asked them to explain some interesting statements that emerged from the discussion. All interview sessions were conducted by the first and second authors, and interview notes were taken for data collection.

### 4.2. Results

Data from observations and interviews were reviewed and summarized into transcripts for thematic analysis [66]. Based on the logical closeness, segmentations of these transcripts were clustered and validated into different themes. In what follows, we present the main findings from our observations of the experience sessions and the follow-up interviews.

#### 4.2.1. Observations

Based on our observations on participants’ interactions with the LightSit system, we found their user experiences could be divided into three stages: learning, exploring, and engaging. Findings from these three stages showed the benefits and challenges of the “LightSit experience” and are reported as follows:

**Learning.** The on-screen user interface of the LightSit software showed instructions about how to interact with LightSit. For some participants, especially the older participants, it was still challenging to start the interaction smoothly. These participants still needed to refer to the demo video to learn the exact working mechanism of LightSit. We observed that, with the assistance of the video, most participants could quickly adapt themselves to using the system to perform slow breathing and lower back stretching exercises. From this observation, we found that the learning cost of LightSit could be maintained at a relatively low level with supportive materials, such as an animated demonstration. LightSit could be easily introduced into real office work with an explicit instruction or user manual for the first use;

**Exploring.** After knowing how to use LightSit, participants could perform the exercise well and started exploring more interactions with the system during the exercises. For instance, many of them tried out different degrees of the stretch and the speed of the posture changes or different slow breathing patterns to control or influence the feedback from the lighting display. For the slow breath training, the HRV biofeedback normally needs about 30 s to get the IBI waveform regulated into the desired state for showing an obvious breathing pattern, which means participants needed to wait some time so that the light pattern could map to their breath. Therefore, at this stage, it happened that a few visitors had a lack of patience for this gradual physiological process;

**Engaging.** In the last stage, we observed that most of the participants could engage in both stretching exercises and slow breathing sessions with the assistance of LightSit. For instance, some participants concentrated on swaying the upper body following the rhythm of the light changes and did not immediately stop the movement after the session. Some participants slightly closed their eyes while breathing to become more relaxed from the exercise. In addition, some participants did not feel one session was enough and wanted to try out the LightSit again. In some cases, they invited their partners or friends with them to experience the system as well. The dynamic patterns from the lighting display also triggered some mutual discussions among participants to share their opinions about our system.

#### 4.2.2. Audience Feedback

According to the follow-up interviews, most participants wished to use LightSit in their own office and wanted to introduce the system to their colleagues and friends, e.g., “Can I find it on the market? I want to buy two, and give one to my boyfriend, to help him with his back pain.” They also explained the reasons, which could be classified into the following three aspects.

First, most of the participants liked the concept of the healthy microbreaks that LightSit is aiming for. They expressed that LightSit provided a new perspective to tackle physical inactivity at work. On the one hand, they thought the idea that interweaving microbreaks and casual desk exercise into office work could bring some benefits to their health: For instance, “I like the way of an incremental gain of my physical health, which is easy for me to learn and keep” and “It encourages me to take small rests with breath and stretch in my work, that helps me to relieve my work pressure”. On the other hand, most participants appreciated the unobtrusive design of LightSit, which facilitates fitness or relaxation breaks for office workers without disturbing them. As one manager expressed from the employer’s perspective, “I know there are some active workstations, which want to help you do exercises while working. But it influences us with productivity. I think my employees would prefer using LightSit. It tells us when to do exercise and offers affordable workplace exercises. So, we can still attach to the work task.”

Second, many participants were impressed by the neat set-up of the LightSit system. They indicated that the unobtrusive sensor embedded into the chair and the ambient lighting display under the stand could be a practical, engaging design for office settings: For example, “I didn’t know there are some sensors in the chair, as it is very comfortable to sit on it, and I don’t feel anything!” After we explained that all of the sensors of LightSit were packaged into one portable seat pad and the display was just an LED strip, some participants also saw potential value in this lightweight feature. As one flex worker stated, “Now more and more people are working like me. We need to work in different spots throughout the week. This technology is easy for me to carry and set up. So, I can always take care of my health no matter where I am”. Additionally, most participants appreciated the ambient lighting display for office vitality, e.g., “The light will just gently decorate my workstation, and it will also play a big role in helping me with my health improvement”. Many of them stated that compared to on-screen displays such as a pop-up window, the changes in light could be both an unobtrusive hint for prompting a break and also dynamic guidance for assisting with exercise. For instance, one participant stated, “The light is hidden under the stand and reflected on the surface of the desk, this soft effect is efficient and work-friendly.”

Third, we found that real-time lighting guidance and feedback for facilitating fitness microbreaks was new and appealing to our participants. The light display that was integrated into the monitor stand could be effective in engaging users in performing stretching exercises and deep breathing. As some of them indicated, “The changes of the light seduced me to follow it, and I did, then I immersed into the exercises,” and “I enjoy stretching my body and taking a deep breath to control the dynamic light.” Some participants also mentioned the potential of LightSit to change their working behaviors in the long term. For example, “When I become more familiar with the interaction, I can actually interact with the light without looking at it. Then, I can also do some light task or chat with my coworkers while exercising,” and “I would like to use it for performing low-back stretch during my work, as I like swaying my upper body when I want to come up with some creative idea”. Based on their comments, we found that the unobtrusiveness of LightSit can be further leveraged as a persuasive feature to encourage behavior changes for workplace health promotion.

## 5. Discussion

This paper presents the design and evaluation of LightSit, a health-promoting system that can prompt and facilitate fitness and relaxation microbreaks in office work. LightSit was designed to support office vitality with two design considerations. First, we embedded sensing and HCI technologies into an office environment to monitor office workers’ sedentary behavior and chronic stress in an unobtrusive manner. Second, we explored leveraging ambient interaction technology to facilitate at-the-desk fitness and relaxation exercises for blending microbreaks into a work routine. A user study was conducted by following the showroom approach to understand the user experience of LightSit-enabled health microbreaks and evaluate its applicability to workplace health promotion. We collected observation data from more than 500 experience sessions and interview data from on-the-spot in-depth interviews with 50 office workers. Our analysis of the observational data showed that the LightSit system provided users with positive interactive microbreak experiences in terms of low learning costs, freedom for exercise exploration, and engagement in health-promoting activities. The results of the interviews revealed a high acceptance of using LightSit for increased vitality in office work, as participants believed the unobtrusiveness, light weight, and interactivity of the system could effectively blend health promotion into the workday context. Based on these insights, we further derived several implications for the design and research of workplace health-promoting technologies.

### 5.1. Design Implications

First, workplace health-promoting technologies should be designed to facilitate microbreaks and interweave desk exercises into a work routine. Most participants thought the types of short and low-effort exercises provided by LightSit were very suitable for refreshment or relaxation during a work break. Based on the ambient lighting display, LightSit could also enable office workers to perform health-promoting exercises during work. Some mentioned that LightSit might make their work break occur more frequently because they could easily access the program and start the exercises quickly by following the guidance from the ambient light under the monitor stand. Compared to a fitness promotion program that requires office workers to stop ongoing work and leave the workstation, a fitness program based on microbreaks can be easier and more “work-friendly” to use without breaking a workflow.

Second, workplace health-promoting technologies should be integrated into the office environment. Most participants thought LightSit was an attractive health promotion system for office workers because of its unobtrusive sensor in the chair and subtle display through ambient light. By integrating pressure sensors into a seat pad, the office chair became not only a tool that collected health-related data, but also a piece of equipment to facilitate health-promoting activities. By integrating a light strip into a monitor stand, the surface of a desk was turned into an ambient display for presenting health conditions during office work. The subtle color changes of light reminded users of their sedentary time and stress levels at the periphery of their attention, which could interfere less with their ongoing work compared to traditional graphical user interface-based displays.

Third, incentive mechanisms should be introduced into the design of workplace health promotion programs. We found that for most participants, both stretching and breathing exercises were easy to learn and perform well. On the one hand, this low learning curve and low mental effort meets the requirement of microbreaks at work. On the other hand, this simplicity may also lead to boredom and loss of motivation after a few weeks of use. Therefore, to strike a balance, we think one possible solution is to introduce incentive mechanisms into the program. For instance, when users finish an exercise, they can receive a “virtual reward” from the system. Afterward, these “virtual rewards” could be used as credits to exchange for some health-boosting allowances, e.g., a healthy meal, a water bottle, or a fitness club membership. Employers or healthcare providers may help to facilitate such exchanges. In the long term, those extrinsic incentives could help office workers both foster healthy habits at work and intrinsically change behaviors and attitudes toward a healthy lifestyle. Furthermore, previous research has suggested that workplace health promotion can be designed in a collective fitness mode through leveraging peer bonding as a motivational factor [13]. In our study, similarly, many participants mentioned the potential of using the LightSit system as a lightweight social game that enables coworkers in one workspace to cooperate or compete at fitness during breaks. For instance, a group-based health goal and/or a leaderboard related to health behaviors could be implemented in the office based on LightSit.

Besides promoting microbreaks, through analyzing long-term data collected from multiple LightSit devices, the system could understand whether sedentary working conditions among coworkers become overwhelming in a workday. In return, coworkers may be encouraged to leave their workstations and initiate participatory activities at higher fitness levels.

### 5.2. Limitations of the Study

Our study had a few limitations. Although the showroom-based evaluation at the international exhibition allowed us to collect feedback from a wide range of participants, their short interactions with LightSit in an exhibition environment might not have been adequate to reveal the effects of LightSit on health promotion from long-term everyday use in real office surroundings. In various environments, the proposed system might meet new challenges. For instance, whether the light display of the HRV data and sedentary time in a public workplace would cause privacy issues or social pressure is still unknown. Besides, although the effectiveness of the system for assisting with lower back stretching exercises and slow breathing relaxation was evaluated in our previous lab-based control experiments [22,33], the effectiveness of the intervention mode of LightSit on prompting work breaks was not evaluated in this study. In the future, a well-integrated LightSit system will be fully deployed in office environments and evaluated in a long-term field study.

## 6. Conclusions and Future Work

In this paper, we presented LightSit, an interactive health-promoting system for office vitality. During office work, LightSit tracks a user’s posture and heartbeat unobtrusively and informs them about their sedentary behaviors and chronic stress through an ambient light to prompt microbreaks as an intervention. During microbreaks, LightSit presents guidance and feedback through a lighting display, which enables the user to do deep breathing and lower back stretching exercises without leaving an ongoing task. In this way, these fitness and relaxation microbreaks can be blended into a workday context to achieve a potential for health promotion. LightSit was evaluated by using a showroom-based approach at a public exhibition during Dutch Design Week 2018. The results suggest that LightSit has the potential to prompt and facilitate small bouts of desk-based fitness and relaxation exercises with relatively low effort without overburdening office work. Besides, we found that integrating sensing and feedback into a workstation made the LightSit system more unobtrusive, and it interfered less with workflow.

Additionally, this study revealed design implications to improve the current design of LightSit to support long-term motivations of office workers toward health promotion and make systemic impacts in encouraging healthy behaviors. In the future, we plan to investigate a new iteration of LightSit by involving some new incentive mechanisms into the system at both individual (e.g., “challenge and reward”) and social levels (e.g., “collaborative fitness”). We then plan to conduct a longitudinal field study with a large group of office workers to validate the effectiveness of our design in promoting workplace health and explore its benefits in supporting a systemic change toward healthier workstyles.
